# The Role of Humoral Innate Immunity in Hepatitis C Virus Infection

**DOI:** 10.3390/v4010001

**Published:** 2012-01-05

**Authors:** Alexander W. Tarr, Richard A. Urbanowicz, Jonathan K. Ball

**Affiliations:** Biomedical Research Unit in Gastroenterology, School of Molecular Medical Sciences, Faculty of Medicine and Health Sciences, University of Nottingham, Nottingham, NG7 2UH, UK; Email: rich.urbanowicz@nottingham.ac.uk (R.A.U.); jonathan.ball@nottingham.ac.uk (J.K.B.)

**Keywords:** innate immunity, hepatitis C virus, complement, defensin, pentraxin, collectin, mannose binding lectin, ficolin, pathogenesis, fibrosis

## Abstract

Infection with Hepatitis C Virus (HCV) causes chronic disease in approximately 80% of cases, resulting in chronic inflammation and cirrhosis. Current treatments are not completely effective, and a vaccine has yet to be developed. Spontaneous resolution of infection is associated with effective host adaptive immunity to HCV, including production of both HCV-specific T cells and neutralizing antibodies. However, the supporting role of soluble innate factors in protection against HCV is less well understood. The innate immune system provides an immediate line of defense against infections, triggering inflammation and playing a critical role in activating adaptive immunity. Innate immunity comprises both cellular and humoral components, the humoral arm consisting of pattern recognition molecules such as complement C1q, collectins and ficolins. These molecules activate the complement cascade, neutralize pathogens, and recruit antigen presenting cells. Here we review the current understanding of anti-viral components of the humoral innate immune system that play a similar role to antibodies, describing their role in immunity to HCV and their potential contribution to HCV pathogenesis.

## 1. Virus-Host Interactions in the Acute Phase of HCV Infection

Hepatitis C Virus (HCV) infects approximately 170 million people worldwide, resulting in chronic, progressive disease in 80% of infected individuals. Infection is characterized by progressive liver damage, resulting in fibrosis and cirrhosis. In 5% of chronic infections hepatocellular carcinoma develops, necessitating liver transplantation. An acute, self limiting infection occurs in 20% of infections [1]. It is believed that control of infection is determined by the interplay between virus and the host immune system [2]. Greater understanding of the virus-host interactions in resolving infections is required to identify correlates of clearance and is an essential for development of new therapeutic interventions and effective vaccines.

Studies of the early stages of HCV infection are limited by the generally asymptomatic nature of infection soon after transmission. Infections are often undiagnosed until clinical presentation of symptoms. As a consequence much of our understanding of viral kinetics in the early stages of infection comes from experimental infection of primates. Both spontaneously resolving and persistent infections have high viral load in the first few weeks [3]. This results in production of HCV-specific T cells within 2–5 weeks [4,5]. Seroconversion occurs approximately 6–8 weeks after infection [6], following the initial T-cell response. Adaptive immunity is believed to make an important contribution to spontaneous resolution. Clearance has been associated with a strong, broadly-targeted T cell response [5,7,8,9], and the rapid production of broadly neutralizing antibodies [10,11]. However, there is no clear consensus; spontaneous resolution of HCV infection can occur in agammaglobulinemic individuals, [12], and T cell responses are not always correlated with resolving infection [13]. The protective effect of host immunity is likely to be multi-factorial, comprising both adaptive and innate components. Many studies have assessed host humoral adaptive immunity in chronic and spontaneously resolving infections [11,14,15,16]. However these studies have attributed anti-viral properties of sera only to the presence of antibodies. It is likely that this underestimates the contribution of innate factors to spontaneous clearance [17] and protection from re-infection [18]. 

Despite advances in our understanding of the role of adaptive response to HCV infection, much less is known about the contribution of acute-phase immune factors to clearance and the innate humoral defenses that act during chronic infection. The mammalian immune system has evolved both innate and adaptive arms to act co-operatively, protecting against infection and limiting the damage caused by invading pathogens. Innate immunity acts immediately following infection, directing production of pro-inflammatory cytokines and orchestrating presentation of antigens to T- and B cells. There is clear evidence that this interplay is essential in clearing viral infections [19,20]. In HCV infections, spontaneous clearance is associated with IFN-γ production [8,21] and production of proteins associated with antigen processing [22]. However, greater understanding of the interplay between innate and adaptive immunity in HCV infection is required to optimize therapies and vaccine strategies. Here we review the importance of innate humoral immune factors in virus infections and describe the accumulating evidence that this arm of the immune response is important in limiting HCV infection. There is evidence for direct anti-viral properties of some innate immune proteins, as well as indirect evidence inferred by the modulation of innate immune activity by virus-encoded proteins. The evidence for suppression of humoral innate immunity by HCV, and the role of these proteins in HCV pathogenesis are also considered.

## 2. The Role of Innate Immunity in Limiting HCV Infection

The innate immune system has a number of roles in recognition and clearance of viral infections. It contributes to immune surveillance in organ systems and the circulation, directly neutralizing infection [23,24,25] as well as triggering inflammation, opsonizing pathogens, and modulating adaptive immunity [26,27,28,29,30]. Complex interplay occurs between cellular components of innate immunity, including monocytes, dendritic cells, platelets, Natural Killer cells and NKT cells. These cells detect pathogens and contribute to clearance by activating T cells and B cells and by directly degrading pathogens [31,32,33,34]. 

The function of innate immune cells is intimately linked with recognition by humoral innate immune proteins, a diverse group of proteins that act as Pattern Recognition Receptors (PRRs). These proteins recognize Pathogen-Associated Molecular Patterns (PAMPs) on the infecting virus particles, or presented on the surface of infected cells. These PRRs include pentraxins, and defense collagens such as C-type lectins and ficolins. In addition, elements of the complement cascade are responsible for recognition of pathogens, both directly and as part of immune complexes consisting of immunoglobulins cross-linked by viral antigens on the surface of virions or infected cells. The complement system and associated pathways also have functional effector properties, activating cascades of proteins responsible for opsonisation and lysis of enveloped virions and infected cells. Humoral innate proteins circulate in the bloodstream, and function both in the serum and in tissues.

The innate immune system is highly integrated with adaptive immunity. Soluble innate molecules can modulate antigen presentation [35,36,37], directing the specificity of T cells and antibodies. In turn, antibodies can trigger and modulate innate antiviral effector mechanisms, contributing to enhanced antigen presentation [38]. Humoral innate immune factors are produced by a number of cell types, including monocytes, lymphocytes, and hepatocytes. Importantly, hepatocytes are the primary source of the complement components, mannose binding lectin, and the ficolins L-ficolin and H-ficolin. Localized high concentrations of these proteins may accumulate in the liver and as such may have important anti-HCV activity at the site of replication. Infection of these cells with HCV might also result in increased production of soluble PRRs [39]. Greater understanding of the interplay between components of the innate immune system acting in the liver may reveal novel therapeutic targets.

## 3. Induction of Acute Phase Proteins in Virus Infections

**Figure 1:**
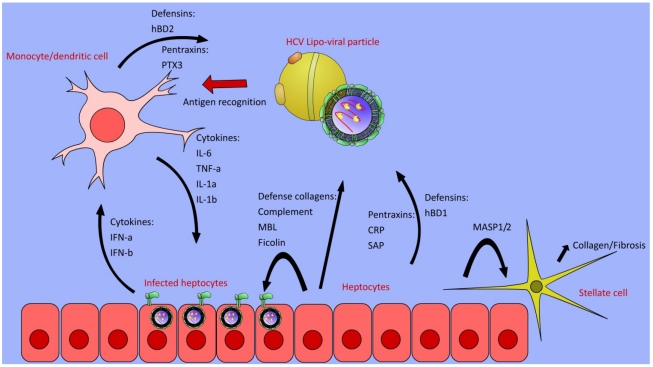
Overview of the production of soluble innate proteins involved in innate recognition of Hepatitis C Virus (HCV) antigens. Infected cells produce type I interferons, supporting activation of the inflammatory response. Antigen presenting cells such as monocytes and dendritic cells also recognize viral antigens, resulting in activation and production of defensins and pentraxins, as well as pro-inflammatory cytokines IL-6, TNF-α, IL-1α and IL-1β. These cytokines induce production of acute phase proteins in hepatocytes such as complement components, pentraxins, and defensins, which contribute to clearance of viruses and infected cells. The hepatocytes are also a source of the defense collagens mannose binding lectin (MBL), and L & H ficolins. These proteins recruit MBL‑associated serine proteases (MASP-1 & MASP-2), triggering the complement cascade and also activating liver-resident stellate cells that produce collagen, resulting in progressive fibrosis.

Initiation of a protective adaptive immune response to HCV infection follows inflammation and as such is dependent on an effective acute phase innate immune response. The inflammatory response is initiated by the presence of virus particles and subsequent infection of hepatocytes. Binding of microbial PAMPs, such as HCV glycoproteins, to antigen presenting cells results in activation and initiation of the inflammatory response and presentation of viral antigens to T cells [40,41,42,43,44,45]. Activation of these cells by viral proteins results in production of the soluble cytokines IL-1α, IL-1β and TNF-α, and the IL-6 family of cytokines, which in turn stimulate hepatocytes to produce acute‑phase proteins. These proteins are classified as Class I or Class II. Class I proteins include complement components, C-reactive protein (CRP), Serum Amyloid A, and α1-acid glycoprotein. These are induced by the action of IL-1α, IL-1β and TNF-α. Class II proteins are induced by IL-6 produced from macrophages, and include fibrinogen, haptoglobin, α1-antichmotrypsin and α1-antitrypsin (reviewed in [46]). These acute phase proteins have roles in hemostasis, increased phagocytosis, and antithrombotic pathways, helping regulate inflammation while mediating clearance of the pathogen. Some of these proteins have a role in pathogen detection, and combine with the constitutively circulating PRRs to recognize pathogens and recruit cells for priming of adaptive immunity (Figure 1).

## 4. Complement Cascade

Complement is central to innate humoral immunity, interacting with a host of soluble and membrane proteins. In addition to the anti-bacterial activity of the complement cascade, this collection of acute phase proteins (C1q, C1r, C1s, and C2-C9) has a spectrum of antiviral activity. Complement components contribute to clearance of virus infections both directly and indirectly, contributing to lysis of enveloped virions [47,48] and virus infected cells [49,50], through the action of the C5-C9 membrane attack complex. Many of the intermediate products of the complement cascade also act as potent opsonins, recruiting cellular immunity to phagocytose antigens and present viral peptides to CD4^+^ T cells. Innate immune cells including monocytes, macrophages and dendritic cells possess receptors CR1, CR2, gC1q-R, and cC1q-R, which bind to the complement/pathogen complexes and process them for activation of adaptive immunity.

**Figure 2:**
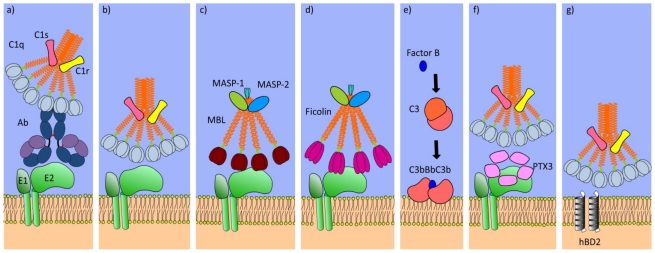
Mechanisms of recognition of viral pathogens by humoral innate proteins that result in complement activation. The widely recognised paths of complement activation include recognition of viral glycoproteins (in the case of HCV, glycoproteins E1 and E2) by: (**a**) antibody (Ab)-mediated C1q binding; (**b**) direct C1q binding; (**c**) mannose binding lectin (MBL); (**d**) ficolins; as well as (**e**) direct deposition of C3b on the surface of viruses or virus-infected cells following activation by Factor B. Additional mechanisms for complement activation by viruses are (**f**) pentraxin (PTX3) binding; and (**g**) binding of C1q to the human β-defensin HBD-2 inserted into a membrane. Complement activation by these recognition molecules is mediated by proteases that cleave the C4 protein. For C1q, these proteases are C1s and C1r. For MBL and ficolins these enzymes are MASP-1 and MASP-2. These ultimately result in C3 cleavage and generation of a C5 convertase complex. C3 cleavage can also occur spontaneously (e), resulting in C4-independent activation of C5. These diverse complement activation mechanisms highlight the broad range of pathogens that can be recognized and eliminated by the complement cascade.

**Figure 3:**
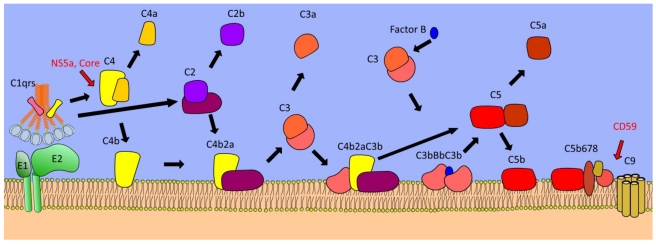
Components of the complement cascade. Initial recognition of pathogens occurs via pattern recognition molecules such as C1q (pictured), mannose binding lectin (MBL), antibodies, and ficolins. Association with serine proteases, such as C1r and C1s result in cleavage of C4 and C2, with the resultant C4b2a membrane-bound complex cleaving C3. Addition of C3b to the C4b2a complex results in the C5 convertase complex C4b2a3b. The Alternative pathway of complement pathway is activated by cleavage of C3 by Factor B. This results in deposition of the C3bBbC3b complex on pathogen membranes, an alternative C5 convertase. Cleavage of C5 results in deposition of C5b, recruitment of C6, C7, C8, which modulate the formation of the membrane attack complex made of oligomeric C9 protein. This causes lysis of enveloped virions, or virus-infected cells. The soluble cleavage products C4a, C2b, C3a and C5a are potent opsonins, recruiting monocytes, neutrophils, and triggering inflammation. Hepatitis C Virus (HCV) interferes with the complement cascade at discrete steps. Both NS5a and Core proteins reduce the activity of C4. In addition, virus particles incorporate CD59 to prevent oligomerization of C9 on the surface of virus particles.

Complement activation by virus infection occurs by five discrete mechanisms (Figure 2). Firstly, the C1qrs complex binds to antibodies captured by either virus-infected cells or virions. Co‑localization of antibody molecules on an antigen results in binding of a bridging complex containing the multimeric recognition protein C1q, This recruits the serine proteases C1r and C1s, which catalyze cleavage of the C4 protein into C4a and C4b subunits [51]. The second mechanism of action is direct binding of C1q viral surfaces, in the absence of immunoglobulin [52,53]. Thirdly, complement is activated by the lectin pathway. The family of ficolins and MBL both bind to glycosylated proteins, recruiting MBL-associated serine proteases (MASPs) to the surface of pathogens, which subsequently cleave the C4 protein [54]. Cleavage of C4 is a central event in the complement cascade, leading to sequential cleavage of C2, The complex of the cleavage products C4b and C2a form a C3 convertase. C3 is cleaved into C3a and C3b (Figure 3) [55]. The C3 cleavage product C3b is deposited on the surface of the pathogen. The fourth mechanism of activation is the spontaneous deposition of C3b onto virus particles. This is independent of recognition of pathogens by PRRs, as C3 is cleaved at a steady rate in the circulatory system by the combination of the activated form of Factor B (Bb), and Factor I. Deposition of the C3b component, either by C2 cleavage or spontaneous deposition, happens on the surface of virions and virus infected cells. C3b has multiple antiviral activities. It acts as a potent opsonin, recruiting antigen presenting cells to the viral antigen. It can also neutralize virus infectivity by aggregating virions. In addition, C3b in complex with either C4b2a, or Factor Bb forms the C5 convertase complex. This leads to deposition of C5b, C6, C7, C8, and C9 proteins on a membrane, generating a membrane-puncturing pore. Other complement components have pro-inflammatory properties. The soluble cleavage products C3a and C5a also initiate inflammation [56], resulting in recruitment of monocytes and neutrophils.

The complement cascade is tightly regulated at every stage of activation, to prevent non-specific action of complement components, protecting host cells from lysis and autoimmune attack. A principal regulator of complement is Factor H, which acts as a co-factor for the C3bBb. This protein binds to host glycosaminoglycans, inhibiting the action of the C3 convertase. The activity of C3b is also regulated following degradation into iC3b and C3d by Factor I. Complement deposition on host cells is inhibited by expression of the membrane proteins CD55 (Decay Accelerating Factor) and CD59 (Protectin), which inhibit the deposition of the C3bBb complex, and the C9 membrane attack complex, respectively. In addition, the host membrane protein CD46 possesses cofactor activity for Factor I, which inactivates both C4b and C3b.

The importance of complement to Flavivirus infections has been revealed by *in vivo* studies of West Nile Virus (WNV) infection. Deficiencies in C3, or complement receptors CR1 and CR2, result in increased WNV pathogenesis [57]. This protective effect is independent of C5a [58], suggesting a role for opsonisation in limiting pathogenesis. Both classical and lectin-mediated complement activation pathways contribute to protection [58,59]. Recruitment of complement component C1q can also modulate the stoichiometry of antibody neutralization, increasing the potency of specific monoclonal antibodies [60]. Despite these advances in understanding the pathology of WNV, there are still only limited investigations of the role of complement components in HCV infection. Complement enhances the neutralization of HCV pseudotypes by antibodies [61]. Although this finding has yet to be confirmed using natural HCV virions, it is plausible that results similar to those for WNV particles will be observed. Complement activation is observed in chronic HCV infection, but with reduced C4 activity [62] and concentration [63]. C4 may play a key role in HCV infection, as both HCV core and NS5A proteins cause reduction in C4 production by inhibiting transcription of C4 mRNA [63]. Greater C4 activity in HCV infections was also associated with better response to standard HCV treatment [62]. This implicates the complement pathway in resolving infection. 

Indirect data also supports the hypothesis that complement has a role in protecting against HCV infection. A recent report suggests that HCV might incorporate CD59 into virus particles [64]. CD59 plays an essential role in preventing complement-mediated lysis of host cells. Present on most cell surfaces, CD59 binds to the C5b678 complex, preventing accumulation of the C9 proteins that form the membrane attack complex. Other viruses incorporate CD59 into their virions, including HIV-1, HTLV-1 and Vaccinia Virus [65,66,67]. It is plausible that HCV lipo-viral particles incorporate CD59 as a mechanism to prevent complement-mediated lysis of HCV virions. The contribution of complement to direct lysis of HCV virions, however, remains to be demonstrated.

The action of complement is modulated by complement receptors that bind C1q. The receptor gC1q-R is a multi-ligand binding protein expressed on the surface of monocytes and macrophages, as well as released from the surface of cells in soluble form. Protein gC1q-R has specificity for the globular head of the C1q molecule [68], as well as vitronectin and thrombin [69]. HCV core interacts directly with gC1q-R protein, resulting in reduced activation of T cells. Soluble gC1q-R is also found in serum in complex with core. Core protein mimics the structure of C1q sufficiently so that cross‑reactive antibodies are produced during infection [70]. This implicates core in sequestering complement components and in preventing activation.

## 5. Defense Collagens

Complement component C1q belongs to the family of defense collagen proteins. The complement cascade can also be activated by other members of this family, specifically the mannose-binding lectin (MBL) and the human ficolins, M-ficolin, L-ficolin and H-ficolin. In contrast to antibodies, these defense collagens have binding specificity for PAMPs rather than epitopes. Other defense collagens are expressed in humans, notably surfactant protein A and surfactant protein D. However, these will not be discussed as they are solely expressed in the lungs, do not activate complement, and function in opsonizing respiratory pathogens.

*Mannose Binding Lectin*. Mannose (or mannan) Binding Lectin belongs to the collectin family of proteins, defined by the presence of a lectin binding domain at the C terminal end of the protein. It is an acute phase soluble protein produced by hepatocytes, with a structure similar to that of C1q. The structure has an N-terminal Cysteine-rich domain, followed by a collagen domain possessing the common Gly-Xaa-Yaa amino acid repeat. At the C-terminus is the lectin binding domain, a structure shared with defense molecules isolated from mammals, amphibians, and plants [71]. The widespread distribution of these proteins in diverse species highlights a conserved evolutionary requirement for this protein. Indeed, absence of MBL in humans is associated with increased susceptibility to opportunistic infections [72,73]. MBL has binding specificity for patterns of mannose and *N*‑aceytlglucosamine [74] associated with pathogen surfaces and apoptotic cells [24,75,76]. MBL possesses similar activity to the C1q protein, resulting in cleavage of C4 and C2 following interactions with the MBL-associated serine proteases (MASPs) [77,78]. Cleavage is inhibited by C1q inhibitor [79]. Humans possess three MASPs, and another similar molecule, MAp19, which does not possess serine protease activity. They have structural similarity to C1s, and cognate roles in activating the complement cascade. MASP-2 has substrate specificity for C4 and C2, resulting in generation of the C3 convertase C4b2a. It also has Factor Xa-like activity, cleaving pro-thrombin to thrombin and initiating the clotting pathway [79,80] MASP-1 cleaves C2, also contributing to complement activation [81]. In addition MASP-1 has been shown to have thrombin-like activity that results in cleavage of fibrinogen and Factor XIII [82,83]. MASP-1 also enhances inflammation by activation of the Protease Activated Receptor 4 (PAR4)-mediated NF-κB pathway and the p38 MAPK activation pathway in endothelial cells [84]. This results in production of pro-inflammatory cytokines, recruitment of leukocyte migration and adhesion. MASP-3 is an alternative splice transcript of MASP‑1, possessing alternative specificity for the insulin-like growth factor binding protein 5 (IGFBP5) [85]. Its role in inflammation and clotting is still not clear. However, the activation of the clotting pathway has implications for deposition of fibrinogen in the site of infection, and may contribute to fibrosis, which is a hallmark of chronic HCV infection [86].

The gene encoding the MBL protein (MBL2) is known to possess polymorphisms that have a significant impact on oligomer formation and serum concentrations of the protein [87,88,89,90,91]. Early investigations of polymorphisms in the MBL2 gene in HCV infections associated underlying genetic differences with treatment outcome [92,93] and disease progression [94]. However, the role of MBL in HCV disease progression is not clear. Some studies have associated low serum levels of MBL to increased chance of liver fibrosis [95], while others demonstrated no effect of serum MBL concentration on HCV disease [96]. In contrast, others associated increased MBL and MASP-1 levels with pathogenesis [86]. Comparison between these studies is complicated by differences in population ethnicity, treatment status, and HCV genotype. Further studies of the activation of inflammation by MBL are required to demonstrate an association with HCV pathogenesis. 

A recent study demonstrated that MBL binds to the HCV surface glycoproteins E1 and E2, activating the complement pathway and also neutralizing entry of HCV into hepatoma cells in a similar way to antibodies [24]. This is consistent with the neutralizing activity of MBL observed against HIV‑1 [23]. Dependent on the viral genotype, HCV E1 and E2 glycoproteins possess between 13 and 15 glycosylation sites that present mannose targets to MBL [97]. These glycosylation sites are proximal to receptor binding sites that are essential for HCV entry. As such, the neutralization of entry mediated by this lectin is consistent with the location of these mannose moieties. This suggests that clinical administration of MBL may be therapeutically useful against HCV infections. However, the pro-inflammatory nature of MBL-associated serine proteases would have to be taken into account when considering passive immunotherapy. Another clinical application for administration of MBL could be intervention during liver transplantation. Serum MBL levels are predictive of susceptibility to nosocomial infection during transplantation [98]. Passive administration could have the effect of reducing infection of a transplanted liver, while at the same time protecting against re-infection of the graft with HCV.

*Ficolins*. The family of ficolins share great structural similarity to the collectins, and play a similar role in innate immunity. Three human ficolins have been identified. M-ficolin (ficolin-1) is produced by macrophages in response to pro-inflammatory cytokines. L-ficolin (ficolin-2) and H-ficolin (ficolin-3, or Hakata antigen) are both expressed by hepatocytes [99]. They share a common overall structure, possessing a Cysteine-rich N-terminal domain, followed by a collagen repeat region. However, in contrast to MBL and C1q they possess a fibrinogen-like head region. The ficolin monomers associate to form higher-order oligomers with high avidity binding properties. All three molecules share binding specificity for acetylated compounds through interactions with the fibrinogen-like domain. Virus glycans often incorporate *N*-acetyl glucosamine (GlcNAc) moieties, which are recognized by ficolins [100]. Each of the glycans associated with the HCV glycoproteins contain a core GlcNAc_2_ moiety, and *in vitro* expression of these proteins has revealed that two of these glycans (N423 and N430) possess terminal GlcNAc residues [97]. It is still unclear if the glycoproteins incorporated into the virus particles possess these specific modifications. However, this hypothesis is supported by the finding that L-ficolin has specificity for the HCV E1 and E2 glycoproteins [39,101], resulting in neutralization of HCVpp and HCVcc [101], and contributing to activation of the complement cascade [39]. Expression of L-ficolin is also up-regulated in HCV-infected cells [39], suggesting that they might constitute a component of the acute phase response following infection. Polymorphisms in the promoter and coding regions of the ficolin proteins are associated with differential expression of L‑ficolin [102], but less variability is observed in serum concentrations when compared to MBL. The indirect functions of ficolins are very similar to MBL. They bind to MASP-1 and MASP-2, triggering complement activation and cleavage of clotting factors in an identical way to MBL [103]. As such, they represent an additional innate PRR with a unique pattern of specificity, broadening the activation profile of the complement cascade. Further investigations are required to establish the role of ficolins in HCV infection. Interestingly, ficolins might be a component of the response that results in treatment induced clearance of infection. M-ficolin is one of only three genes that display up-regulated expression in individuals with the protective CC allele of the rs12979860 polymorphism in the IL28B promoter region [104]. This allele is associated with favorable treatment response and disease outcome [105]. This contrasted with lower expression of the pathway of interferon-stimulated genes in patients with this IL28B variant allele. It is plausible that increased M-ficolin expression represents a functional consequence of this polymorphism, resulting in enhanced neutralization, opsonization, or complement deposition required to suppress virus infection. However, further studies are required to extend these preliminary findings. 

## 6. Pentraxins

Pentraxins are a conserved family of pattern recognition molecules produced in genetically diverse species, suggesting a strong selective advantage for these proteins. Members of this family share structural homology, with a conserved 200aa C-terminal pentraxin domain [106]. This diverse group includes the short pentraxins Serum Amyloid P (SAP) and C-reactive protein (CRP), and the long pentraxins exemplified by PTX3. Short pentraxins are acute phase proteins produced in the liver [107], while long pentraxins are expressed by dendritic cells in response to activation of TLR signaling pathways [108]. Despite structural differences, all pentraxins are pentameric protein structures with calcium-dependent binding to lipoproteins, as well as calcium-independent binding to other ligands. CRP is an acute phase protein that is produced by hepatocytes in response to IL-6. It activates complement C1q in the presence of phosphocholine, produced by apoptotic cells and present in the capsule of some bacterial species. Although it is believed not to be involved in direct recognition of viruses, CRP has been implicated in mixed cryoglobulinemia associated with HCV infection [109]. More relevant to protection against virus infections is the prototype long pentraxin PTX3 [110]. It has diverse binding interactions, including host proteins, bacteria, and virus glycoproteins [111,112]. Like CRP, PTX3 also binds to the globular head domain of complement C1q. This interaction augments complement activation when PTX3 is immobilized [113], but inhibits antibody interactions with C1q when in solution. This suggests that PTX3 binding to virus glycoproteins might provide an additional mechanism by which complement can be deposited on viruses and infected cells, enhancing deposition of C1q and C3b on apoptotic cell surfaces. PTX3 is a rapid acute phase protein. Expression is immediately up-regulated following infection or inflammation [114]. PTX3 is recruited to the synapse between dendritic cells and apoptotic cells. This results in modulation of antigen presentation pathways in the DC, reducing the cell’s cross-presentation of HCV antigens to CD8^+^ T cells [115]. It remains to be determined if this pattern recognition molecule has a role in the immune response to HCV infection.

## 7. Lipoproteins

There is an emerging role for lipoproteins in innate immunity. Serum Amyloid A1 (SAA-1) and Serum Amyloid A2 (SAA-2) are acute phase lipoproteins rapidly produced in the liver following trauma or infection, or following stimulation by the inflammatory cytokines IL-6 and TNFα [116,117]. The SAA proteins potently enhance cytokine production from peripheral blood mononuclear cells [118], promoting inflammation. In HIV-1 infection, SAA production occurs significantly before detection of viral RNA [17], suggesting that this may have a significant role in limiting the initial viral burst of replication. As well as its role in acute phase inflammation, SAA plays a physiological role in transport of cholesterol mediated by Scavenger Receptor B1 (SR-B1) [119,120,121]. SAA circulates as complexes with high-density lipoprotein (HDL) and causes remodeling of HDL in acute phase inflammation. As such, SAA may play a unique and intriguing role in preventing HCV infection. SR-B1 and HDL are implicated in the entry pathway of HCV. SR-B1 binding of virions is essential in the early steps of HCV entry [122,123,124,125], and this entry is augmented by binding of HDL [126,127,128]. SAA inhibits HCV entry by binding to virus particles [129,130], and this interaction is inhibited by the presence of HDL [129]. Together these findings suggest that SAA binds to HCV lipo-viral particles (LVP) consisting of lipoproteins and the HCV virion, blocking the interaction between LVPs and SRB1. In this model, HDL is able to strip away SAA from particles and enhance entry of the virus into hepatocytes. It is unclear if this anti-viral pathway has *in vivo* efficacy. Studies of the circulating SAA concentrations in chronic HCV infection have shown slightly increased levels [129], or no difference to normal controls [131]. This contrasts with acute viral infections, and is consistent with the observation that SAA is produced only in the early stages of acute infection [131]. 

In addition to SAA, high-density lipoproteins may also have a role in innate immunity. Along with the major role of HDL in promoting cholesterol efflux from peripheral tissues and recovery in hepatocytes, HDL modulates activation of the complement cascade, as well as influencing expression of PTX3 [132]. This implicates HDL in orchestrating innate immunity in the liver, modulating the action of soluble innate factors.

## 8. Defensins

Humans express two types of defensins, α-defensins (human neutrophil peptides HNP1-4, HD5 and HD6), and β-defensins (hBD1-4). Both types of defensin are short (20–40 amino acids) soluble, charged peptides with anti-microbial properties [133,134]. The α-defensins possess anti-viral activity, blocking virus entry and replication [25,135], as well as modulating complement activation by C1q [136]. They act by inserting into membranes, destabilizing virus particles and recruiting macrophages and enhancing phagocytosis. The β-defensins play a similar role in antiviral defense, binding to virions and preventing entry [23]. hBD-1 is constitutively expressed by most cell types, but expression can be up-regulated in plasmacytoid dendritic cells (pDCs) and monocytes in reaction to virus infections [137]. In contrast, hBD2 is an acute phase protein produced by pDCs, monocytes, and epithelial cells in response to inflammatory cytokines and the presence of virus infection [138]. hBD2 can bind C1q resulting in complement deposition [139]. hBD have been implicated in protection against HIV-1 infection in serially exposed uninfected individuals [140]. Despite the broad range of anti-viral activity observed for defensins [141] there is a paucity of research investigating the role of defensins in HCV infection. The α-defensins are produced in chronic HCV infection, and correlate with liver fibrosis [142]. However, it is unclear if binding of these peptides to virus particles inhibits infection *in vitro* or *in vivo.*

## 9. Evasion of Immune Responses

HCV utilizes a number of mechanisms to evade host defenses, including elements of the humoral innate immune system (Table 1). The majority of virions circulate in the blood associated with lipoproteins [143,144], forming a lipo-viral particle [145]. This complex includes the HCV virion, and low density lipoproteins containing apolipoproteins E, C1 and B [146,147]. These associations are mediated by the HCV-encoded glycoproteins and are intimately related to resistance to antibody neutralization [148,149]. HCV virions are also extensively glycosylated, which is believed to reduce the immunogenicity of the envelope glycoproteins and contribute to evasion of recognition by neutralizing antibodies [150,151]. Additionally, three hypervariable regions in the E2 protein [152] tolerate extensive genetic heterogeneity and act as immune ‘decoys’, possessing immunodominant epitopes to which antibodies are rapidly produced [153]. Despite these evasion mechanisms, targets for innate immunity remain conserved; the glycosylation motifs that are essential for HCV biogenesis represent a target for defense collagens. Many of these glycans are conserved between functional isolates representing all HCV genotypes [152]. If these innate binding proteins have a neutralizing effect on virus replication, mutants escaping recognition would be expected to be preferentially selected. To date, defense collagen-resistant HCV strains have not been described. Some glycosylation sites in the envelope glycoproteins do vary between isolates, suggesting at least some plasticity in the glycosylation surrounding virus particles. There is evidence from pandemic H1N1 influenza viruses that supports the proposition that defense collagens apply selection pressure on virus populations [154]. It is plausible that other innate immune proteins apply selective pressure on the viral population in the early stages of infection following transmission. Longitudinal analysis of the susceptibility of HCV isolates to recognition by innate proteins will reveal if this is a biologically relevant effect. There is evidence of evasion of other innate humoral proteins, especially the complement cascade. The incorporation of CD59 into HCV virions suggests that HCV has evolved to resist deposition of the membrane attack complex onto virions [64]. The inhibition of C4 production by HCV core and NS5A proteins also provides evidence that there is a selective advantage to disabling the complement cascade [63]. A novel, yet undescribed result of the association of virions with lipoproteins may be the prevention of Serum Amyloid A binding to the virus, preventing the inhibitory effects of this innate protein. 

**Table 1:** Hepatitis C Virus (HCV) interactions with components of the humoral innate immune system and possible escape from innate immune recognition.

Component	Function	HCV Escape Mechanism	Reference(s)
Complement C4	Key mediator of opsonisation, direct lysis, and inflammation	Core and NS5a proteins inhibit transcription of C4	[63]
Complement C5-9	Generation of the membrane attack complex	Incorporation of CD59 into HCV virions	[62]
gC1q-R	Enhanced chemotaxis and phagocytosis; decreased activation of B- and T cells	Core interacts with gC1q-R, suppressing T cell activation	[70]
Defense collagens (MBL; Ficolins)	Recognition of glycoproteins	HCV glycoproteins have differential glycosylation patterns; association with lipoproteins may block access of defense collagens	[152]; [145]
Serum Amyloid A (SAA)	Binding to glycoproteins, inhibiting entry	Interaction with High-density lipoprotein (HDL)/SR-B1 prevents SAA binding?	

## 10. Role of Innate Immunity in Pathogenesis of HCV Infection

So far we have established that the humoral innate immune system contributes to immunity against viral pathogens, including HCV. However, these same proteins can contribute to the pathology of HCV disease [155]. Innate immunity is broadly implicated in liver fibrosis in chronic HCV infection, and initiation of cryoglobulinemia that is associated with 40% of HCV infections. Liver fibrosis is caused by the accumulation of extracellular matrix in the liver, particularly collagen. This deposition of fibrotic material is mediated by stellate cells, portal fibroblasts and myofibroblasts (reviewed in [156]). In HCV infection, fibrosis severity correlates with activation of stellate cells [157], which can be activated by cleavage of Protease Activated Receptors by thrombin [158,159]. This implicates MBL-associated serine proteases in stellate cell activation. Indeed, activity of MBL/MASP-1 complexes is positively associated with developing fibrosis [86,95,160,161].

Another pathology specifically associated with innate immunity in HCV infection is the accumulation of autoantibodies, resulting in cryoglobulinemia [162]. These antibodies are directed to a range of host proteins, including immunoglobulins [163], cardiolipin [164], and members of the soluble innate immune proteins. Despite there being no evidence for CRP interactions with HCV, autoantibodies to CRP are produced in HCV infection, related to cryoglobulinemia [109]. Antibodies to C1q are observed in up to 38% of HCV infections [165]. The production of autoantibodies that react with C1q results in vascular damage [166], a pathology associated with mixed cryoglobulinemia. HCV-associated cryoglobulinemic glomerulonephritis is also associated with autoantibodies to the C1q and C4d molecules [167], as well as MBL and MASP [168]. Autoantibody production might be a consequence of the interaction between HCV E2 protein and CD81 on the surface of B lymphocytes. This interaction is known to reduce the activation threshold and promote hypermutation of the immunoglobulin gene [169,170], indicating a mechanism for autoantibody production.

The complement receptor gC1q-R may have an extremely important role in resolution of infection. HCV core protein interactions with gC1q-R can cause pathogenic effects *in vivo*. In chimpanzees, expression of this receptor on the surface of T-cells correlated with reduced capacity for activation and proliferation, suggesting that the core interaction inhibits T cell activation [171,172]. Circulating gC1q-R has also been implicated in mixed cryoglobulinemia. This soluble form of the receptor is caused by dysregulation of gC1q-R shedding following interaction with core protein. The receptor circulates as a complex with core, sequestering the C4d cleavage product to vascular tissue and causing complement mediated lysis [166].

## 11. Contribution of Innate Immunity to HCV Vaccination and Therapy

Designing vaccines to highly mutable RNA viruses poses a significant challenge. It is likely that activation of broad T cell responses, and potently neutralizing antibodies will be required for suppression of viral replication and clearance. However, optimizing antigen recognition by innate immunity is essential to induce the optimal adaptive immune response. Vaccine induced immunity is regulated by stimulation of innate immune factors such as complement and the defense collagens. These innate molecules provide co-stimulatory signals that are required for the generation of protective immune memory. Greater understanding of innate immune modulation will yield directed vaccines synthesised to optimize production of adaptive immunity. The evidence supporting the role of HCV proteins in inhibiting many aspects of innate immunity informs the search for important innate factors. Controlling gene transcription to alter the serum concentration of these humoral proteins might improve vaccine efficacy. There is evidence that complement components have a role in regulating activation of B cells and T cells [173,174,175]. They also augment antibody neutralization [60], and can directly neutralize virus infectivity [176]. This is likely to be the case for additional PRRs that modulate complement activation. These properties might also prove useful in augmentation of therapies by passive administration of soluble PRRs. Direct neutralization of virus particles and indirect immune activation are both desirable qualities. However, given the role of these molecules in activating stellate cells, therapeutic forms of these proteins might need modification to retain anti-viral properties, while preventing collagen deposition. Synthetic, recombinant forms of these lectins have recently been used to improve ligand affinity and activity [177]. Similar modifications might develop therapeutically useful molecules.

## 12. Concluding Remarks and Future Perspectives

There is an emerging role for soluble innate immune proteins in contributing to neutralization of HCV infection and clearance of HCV infected cells. There is evidence that pattern recognition molecules such as MBL, serum ficolins and lipoproteins all contribute to recognition of virus infections. This leads to activation of complement pathways, resulting in specific lysis of virions, opsonization of viruses and increased phagocytosis, and direct neutralization of virus particles. The importance of this anti-viral response is highlighted by modulation of key complement components by HCV-encoded proteins. However, there is still little known about the interplay between HCV and the complement cascade.

The neutralizing effect of these innate molecules is an under-appreciated phenomenon. Many studies of serum neutralization do not account for the presence of these proteins. There are no studies of the dynamics of acute-phase proteins in early HCV infection, and many reports of serum neutralization of HCV entry consider antibodies as sole mediators of neutralization [11,14,15,16]. Studies using fractionated serum resolve the potential for multi-factorial neutralization in HCV infections [178,179]. Greater understanding of the dynamics of pattern recognition receptors and neutralizing antibodies will define the relative contributions of these two interrelated immune effectors.

Little is known about the evolution of HCV in response to PRRs. Although there is evidence that other RNA viruses escape recognition by collectins, there is little evidence of selection of PRR‑refractive HCV isolates, either *in vitro* or *in vivo*. It is possible that the emerging quasi-species following transmission of HCV is shaped by the surveillance PRRs and the acute-phase proteins that are produced immediately following infection. This would make PRRs important therapeutic interventions that might prevent establishment of acute phase viraemia and development of chronic infections. Although under-studied, innate immunity has great potential as a target for the development of novel anti-HCV therapies.

## Conflict of interest

The authors declare no conflict of interest.
